# REPORTING OF ADVERSE EVENTS IN BEHAVIORAL INTERVENTION TRIALS: THE TAILORED ACTIVITY PROGRAM

**DOI:** 10.1093/geroni/igz038.652

**Published:** 2019-11-08

**Authors:** Daniel Scerpella, Danny L Scerpella, Katherine A Marx, Laura N Gitlin

**Affiliations:** 1 Johns Hopkins University Center for Innovative Care in Aging, Baltimore, Maryland, United States; 2 Johns Hopkins University, Baltimore, Maryland, United States; 3 Drexel University, Philadelphia, Pennsylvania, United States

## Abstract

Adverse events (AE) are not regularly reported in behavioral research, especially when compared to similar reporting in pharmacological research. AE reporting is crucial for understanding the potential risks and outcomes of behavioral interventions as well as characterizing the study population. This presentation presents AE findings of a behavioral intervention to reduce neuropsychiatric behaviors in persons living with dementia, the Tailored Activity Program. A total of 250 dyads (persons living with dementia and caregivers) living in a community setting participated in this intervention, reporting 126 adverse events and 37 alerts. None of the events were attributable to the intervention, with the majority (46%) being related to the health of the person living with dementia or high depression scores in caregivers (19%). These results are analyzed in relation to outcomes and the potential frailty of this study population. Reporting AEs in behavioral trials is the best opportunity to inform treatment options.

